# The Eight Unanswered and Answered Questions about the Use of Vasopressors in Septic Shock

**DOI:** 10.3390/jcm12144589

**Published:** 2023-07-10

**Authors:** Olfa Hamzaoui, Antoine Goury, Jean-Louis Teboul

**Affiliations:** 1 Service de Médecine intensive réanimation polyvalente, Hôpital Robert Debré, CHU de Reims Université de Reims, 51092 Reims, France; agoury@chu-reims.fr; 2 “Hémostase et Remodelage Vasculaire Post-Ischémie”—EA 3801, Unité HERVI, 51100 Reims, France; 3 Service de médecine intensive-réanimation, Hôpital de Bicêtre, AP-HP, Université Paris-Saclay, DMU CORREVE, FHU SEPSIS, 94270 Le Kremlin-Bicêtre, France; jean-louis.teboul@aphp.fr; 4 INSERM-UMR_S999 LabEx—LERMIT, Hôpital Marie-Lannelongue, 92350 Le Plessis Robinson, France

**Keywords:** vasopressors, vasopressin, norepinephrine, septic shock

## Abstract

Septic shock is mainly characterized—in addition to hypovolemia—by vasoplegia as a consequence of a release of inflammatory mediators. Systemic vasodilatation due to depressed vascular tone results in arterial hypotension, which induces or worsens organ hypoperfusion. Accordingly, vasopressor therapy is mandatory to correct hypotension and to reverse organ perfusion due to hypotension. Currently, two vasopressors are recommended to be used, norepinephrine and vasopressin. Norepinephrine, an α_1_-agonist agent, is the first-line vasopressor. Vasopressin is suggested to be added to norepinephrine in cases of inadequate mean arterial pressure instead of escalating the doses of norepinephrine. However, some questions about the bedside use of these vasopressors remain. Some of these questions have been well answered, some of them not clearly addressed, and some others not yet answered. Regarding norepinephrine, we firstly reviewed the arguments in favor of the choice of norepinephrine as a first-line vasopressor. Secondly, we detailed the arguments found in the recent literature in favor of an early introduction of norepinephrine. Thirdly, we reviewed the literature referring to the issue of titrating the doses of norepinephrine using an individualized resuscitation target, and finally, we addressed the issue of escalation of doses in case of refractory shock, a remaining unanswered question. For vasopressin, we reviewed the rationale for adding vasopressin to norepinephrine. Then, we discussed the optimal time for vasopressin administration. Subsequently, we addressed the issue of the optimal vasopressin dose, and finally we discussed the best strategy to wean these two vasopressors when combined.

## 1. Introduction

Septic shock is a circulatory failure that can combine different mechanisms such as hypovolemia, vascular tone depression, cardiac dysfunction, and microcirculation disturbances. As different therapies are directed toward these different mechanisms, it is important to identify the severity of each mechanism to individually select the most appropriate urgent treatment. Fluid administration should not be delayed [1]. 

Another important resuscitative therapy in septic shock is administration of vasopressors. Indeed, release of inflammatory mediators during sepsis may be responsible for depressed vascular tone and hyporesponsiveness to vasoconstrictive agents [2]. This results in systemic vasodilatation and arterial hypotension, which induces or worsens organ hypoperfusion when the mean arterial pressure (MAP)—the upstream pressure for most vital organs—decreases below a certain critical level. The importance of this phenomenon in septic shock has been confirmed by studies showing that severe and prolonged hypotension (MAP < 65 mmHg) was independently associated with mortality [3] and that correction of hypotension with norepinephrine improved renal function and decreased hyperlactatemia even in the absence of an increase in cardiac output [4]. The expected benefit of vasopressors is thus to restore organ perfusion in territories where flow is dependent on pressure. On the other hand, the risk of vasopressors is to excessively vasoconstrict some territories and to eventually result in worsening rather than improvement of organ perfusion and function. The benefit/risk balance of vasopressor therapy is thus important to consider before its initiation as well as during its utilization, especially when the predefined endpoints of resuscitation are not yet achieved. The choice of the agent is important to consider in the benefit/risk balance of vasopressor therapy. Today, two vasopressors are recommended to be used, norepinephrine and vasopressin. In this article, we present some resolved and unresolved questions (Table 1 and Table 2) about the use of these two vasopressors in patients with septic shock. 

## 2. Norepinephrine

### 2.1. Why Is Norepinephrine the Recommended First-Line Vasopressor in Septic Shock?

Until the end of the 20th century, norepinephrine, an α_1_-agonist agent, was administered only in the case of hypotension refractory to dopamine, and thus as a last resort, inherently associated with increased mortality [5]. Today, norepinephrine is recommended as the first-choice vasoconstrictor [6,7]. This recommendation was based on the results of a randomized controlled trial (RCT) showing less cardiac arrhythmia with norepinephrine compared with dopamine [8] and on meta-analyses showing less mortality and less cardiac arrhythmia with norepinephrine [9,10]. 

### 2.2. Should Norepinephrine Be Administered Early or Only after Completion of Fluid Resuscitation?

Until recently, there was a common preconception that norepinephrine should be started only after completion of fluid resuscitation in patients with septic shock. A survey, conducted in 2016–2017 for the European Society of Intensive Care Medicine (ESICM) (839 respondents from 47 countries), showed that only 12% of intensivists administered norepinephrine before completion of fluid resuscitation [8]. This result appears surprising since (1) during septic shock, excessive release of inflammatory mediators and endothelial dysfunction result in depressed vascular tone and hence in hypotension, and (2) fluid administration alone may further decrease arterial tone when it increases cardiac output [11]. Indeed, fluid administration may increase cardiac output in preload-responsive patients. In that case, fluids may decrease systemic vascular resistance [11]. This can be related to the decrease in the sympathetic reaction secondary to a low cardiac output and to flow-mediated vasodilation following arteriolar nitric oxide secretion [12]. Thus, in septic shock, it would be logical to start norepinephrine at the same time as fluid resuscitation. How, then, can we explain the reluctance of most clinicians to do so? Firstly, there has been an old belief that, in hypovolemic conditions, norepinephrine might worsen tissue hypoperfusion due to its vasoconstrictive effects on arterioles. However, there is no evidence to support such a belief in septic shock. In this regard, Georger et al., administering norepinephrine early in patients with severe shock, reported a significant increase in tissue oxygen saturation and better recruitment of peripheral microvessels [13]. Secondly, at the starting phase of shock resuscitation, patients are more often equipped with a peripheral venous catheter. For years, clinicians have been reluctant to infuse norepinephrine peripherally due to the risk of drug extravasation into the adjacent subcutaneous tissues, eventually causing local ischemia [14]. However, a recent systematic analysis of 14,385 patients who received norepinephrine peripherally showed that extravasation of the drug was exceptional (five cases) and never required a specific surgical or medical treatment [15]. Accordingly, the current version of the Surviving Sepsis Campaign (SSC) guidelines suggests starting norepinephrine peripherally to correct hypotension, rather than delaying initiation until central venous access is secured [7]. Based on a systematic review of extravasation case reports [14], the SSC experts stated that administration of norepinephrine for a short period of time (<6 h), in a well-placed peripheral catheter proximal to the antecubital fossa, is unlikely to cause local tissue injury [7]. Of note, 34 ESICM experts agreed on a reasonable consensus (70–80% experts agreed) on the use of norepinephrine before completion of fluid resuscitation, a result that was in disagreement with the non-expert respondents [6]. It is likely that, in the near future, the positions among intensivists will be closer, first due to the recent recommendation to use temporarily peripheral catheters for drug infusion (see above), and second due to the publication of studies providing arguments in favor of early norepinephrine administration in septic shock (see below).

There is no doubt that life-threatening hypotension requires urgent correction with a vasopressor [16]. Even if there is no clear definition of life-threatening hypotension, data showed that a MAP staying below 65 mmHg is associated with increased mortality [3], and there is a consensual recommendation to initially target a MAP of at least 65 mmHg [6,7,16,17]. Note that the normal MAP in subjects without any cardio-vascular comorbidity is around 90 mmHg [18]. Knowing that MAP depends essentially on cardiac output and systemic vascular resistance, a MAP < 65 mmHg is necessarily associated with either low systemic vascular resistance or low cardiac output with insufficient compensatory increase in vascular tone. In both cases, it would be hard to achieve rapidly a MAP above 65 mmHg without vasopressor administration. Therefore, during sepsis, a MAP < 65 mmHg associated with signs of shock should prompt administration of norepinephrine before completion of fluid resuscitation. The more severe the hypotension, the more urgent should be norepinephrine administration. In case of any doubt, a simple way is to look at the diastolic arterial pressure (DAP), which is a good marker of vascular tone [19]. In the presence of low DAP (e.g., lower than 40 mmHg), the vascular tone is assumed to be very depressed—especially in cases of tachycardia [20]—so that starting norepinephrine is a reasonable therapeutic option, whatever the volume of fluids already administered.

Further to its α_1_-agonist effects on arterial tone, early administration of norepinephrine can increase cardiac output through two different effects, increase in cardiac preload [21,22] and contractility [23]. First, it can increase the mean systemic pressure—the upstream pressure for venous return—through a redistribution of venous blood from the unstressed to the stressed blood volume in relation to a decrease in venous capacitance (α_1_-agonist effect) [24]. Adding norepinephrine to fluids potentiates the effects of fluid on the mean systemic pressure and thus on cardiac output [25]. Second, norepinephrine can increase cardiac contractility [23] due to its action on the myocardial β_1_-receptors, which are probably not yet down-regulated at the early phase. Another potential inotropic effect of norepinephrine involves its α_1_-agonist action on arterial tone elevating the DAP, which is the upstream pressure of the left ventricular perfusion. This may be important in patients with coronary artery disease. Furthermore, adding norepinephrine may improve ventriculo–arterial coupling [26].

Another argument in favor of early administration of norepinephrine is to reduce the volume of infused fluids [27], which may prevent fluid overload and its harmful effects on organ function and eventually on outcome [28]. Finally, observational and randomized controlled studies showed that early administration of norepinephrine was associated with improved outcome [27,29,30,31].

### 2.3. How Should the Dose of Norepinephrine Be Adapted?

There is a consensus to titrate the norepinephrine dose on the MAP and a value of at least 65 mmHg is usually recommended as the initial target [6,7,16,17]. Below this value, there are high risks of multiple organ hypoperfusion and increased mortality [3]. There is, however, a debate on the optimal MAP to target. Should the optimal MAP target be a little above 65 mmHg (e.g., 65–70 mmHg) in every patient or should it be in a higher range in some categories of patients? The advantage of maintaining MAP in a range of 65–70 mmHg is to avoid excessive doses of norepinephrine and their potential adverse effects. On the other hand, the risk is not to achieve in some patients the critical MAP value below which organ blood flow can be dependent on pressure. Some studies in human septic shock showed no benefit of increasing MAP above 65 mmHg in terms of organ blood flow or microcirculation variables [32,33,34]. Some other studies found opposite results, with better peripheral perfusion or oxygenation with 75 or 85 mmHg compared to 65 mmHg [35,36,37,38]. A large multicenter RCT comparing low (65–70 mmHg) vs. high (80–85 mmHg) MAP targets in patients with septic shock found no difference in mortality and morbidity. However, atrial fibrillation was more frequent in the high-target arm (7% vs. 3%) [39]. Of note, the observed MAP in the low-target group were, for the most part of the study, between 70 and 75 mmHg. Similarly, the observed MAP in the high-target group was often >85 mmHg [39]. Another multicenter RCT, performed in patients 65 years or older receiving vasopressors for vasodilatory hypotension, showed no difference in outcome between a strategy aimed at maintaining MAP between 60 and 65 mmHg—called permissive hypotension—and usual care [40]. Note that, in that study, the MAP at randomization was already above 60–65 mmHg in the permissive hypotension arm (median MAP: 69 mmHg), and the median MAP values after randomization were not very different in the two groups (67 mmHg with permissive hypotension vs. 73 mmHg with usual care). In addition, 30% of patients received metaraminol, a vasopressor that is not recommended in septic shock. These two large RCTs [39,40] showing no major difference between “low” and “high” MAP targets have some limitations. First, as mentioned above, the observed targets between the two arms were too close to expect different outcomes. Second, by design these studies do not allow definitive answering of the question, since some patients randomized in the low-target arm could have benefited more by being managed according to the high-target strategy and vice versa. In any case, the results of these trials cannot be interpreted in favor of achieving the lowest MAP target in every patient as surprisingly suggested by the last version of the SSC guidelines [7]. Today, rather than achieving a fixed MAP target in every patient, it is suggested to individualize the target according to some specific factors [16,17]. For example, in patients with chronic hypertension, the critical MAP below which organ blood flow depends on organ perfusion pressure should be higher than in non-chronic hypertensive patients [41]. Thus, the MAP target could theoretically be higher than 65 mmHg in cases of chronic hypertension. Accordingly, in the study by Asfar et al. [39] benefits in terms of kidney function were observed in the subgroup of patients with chronic hypertension randomized to the high MAP target. This was confirmed in a recent meta-analysis [42]. Otherwise, organ perfusion pressure depends on MAP as the upstream perfusion pressure but also on the downstream perfusion pressure. If the downstream perfusion pressure is elevated, the organ perfusion pressure can be insufficient even if MAP is higher than 65 mmHg. This could occur in cases of intra-abdominal hypertension or increased central venous pressure (CVP). In this regard, Ostermann et al. reported that the mean perfusion pressure (MPP = MAP − CVP), but not MAP alone, was independently associated with the progression of acute kidney injury and found a cutoff value of 60 mmHg for MPP [43]. Furthermore, it was shown that increased CVP was associated with decreased microvascular flow of the sublingual territory [44]. In cases of high CVP, the best option would be to decrease CVP when possible [45]. If the decrease in CVP in not doable, the alternate option is to increase the MAP target to ensure an MPP > 60 mmHg [43].

### 2.4. Should We Escalate the Doses of Norepinephrine in Cases of Refractory Hypotension?

Hyporesponsiveness to vasoconstrictors, especially to α_1_-agonist agents is a hallmark of septic shock [46,47]. In severe cases, usual doses of norepinephrine can be ineffective to totally restore arterial tone. This situation may occur when the source of sepsis is not well controlled, and must be checked. Otherwise, there are three options: (1) continuing escalating the doses of norepinephrine, (2) administering intravenous (IV) hydrocortisone, or (3) adding another vasopressor. Continuing escalating the doses of norepinephrine exposes to the risks of serious adverse effects such as myocardial cell injury, alteration of sepsis-associated immunomodulation, and mesenteric, limb, and digital ischemia. Serious adverse events more often occur in the most severe patients [48]. Retrospective data showed that receiving more than 1 µg/kg/min norepinephrine is associated with a high risk of death [49], although contradictory results were also reported [50]. Recent data showed that the in-hospital mortality rate dramatically and continuously increased as the maximum norepinephrine equivalent dose in the first 24 h of shock onset increased, even after adjustment for the patient baseline characteristics and the severity score [51]. Therefore, the option to escalate the dose of norepinephrine without trying another pharmacological option is no longer recommended [7]. Administering IV hydrocortisone is a reasonable alternate option. It is now suggested to commence it at a dose of ≥0.25 mcg/kg/min at least 4 h after initiation [7]. The expected advantage of hydrocortisone is to potentiate the vasoconstrictive effect of norepinephrine on the vascular α_1_-receptors. This can increase MAP [52,53], especially in cases of impaired adrenal reserve [52]. Previous data showed a more rapid shock reversal with hydrocortisone compared to placebo [54,55,56]. Another option is to administer a second vasopressor. The SSC suggests adding vasopressin instead of escalating the dose of norepinephrine [7].

## 3. Vasopressin

### 3.1. What Is the Rationale for Adding Vasopressin to Norepinephrine in Some Forms of Septic Shock?

Vasopressin (arginine–vasopressin, AVP, also called “antidiuretic hormone” ADH) is a natural hormone released from the post-pituitary gland [57]. The rationale for using vasopressin in septic shock is based on pathophysiological mechanisms and clinical data. Vasopressin induces vasoconstriction, after binding to V1a receptors, which are located on vascular smooth muscle cells [58]. In septic shock, circulating levels of vasopressin were found to be abnormally low with respect to the degree of hypotension [59,60,61]. Administration of exogenous vasopressin makes sense since there is a relative deficiency in circulating endogenous vasopressin in septic shock [62]. Administration of exogenous vasopressin reduces norepinephrine requirements [63], and thus might prevent occurrence of β-agonist-related adverse events including dysregulation of the immune host response to sepsis [64,65] and myocardial toxicity [66,67,68]. Indeed, excessive doses of catecholamines may cause sympathetic overstimulation, leading to detrimental cardiac effects including impaired diastolic function, tachycardia and tachyarrhythmia, myocardial ischemia, stunning, apoptosis, and necrosis.

In addition, unlike norepinephrine, which increases resistance in both afferent and efferent glomerular arteries, vasopressin increases resistance only in the efferent arterioles [69]. It is even possible that by activating the V_2_ receptors, vasopressin might vasodilate the afferent glomerular arterioles [70]. Both effects may increase glomerular pressure and flow. Accordingly, in a randomized and double-blind study comparing norepinephrine and vasopressin in severe septic shock, only vasopressin was able to increase urine output and creatinine clearance, and these effects were not related to increase in MAP or in cardiac output [71]. The VANISH trial compared, in patients with septic shock, norepinephrine alone and early vasopressin added to norepinephrine within a maximum of 6 h after the onset of shock [72]. Early use of vasopressin did not increase the number of kidney failure-free days and mortality rates were similar between all groups [72]. There was, however, a lower rate of renal replacement therapy requirement with vasopressin [72]. An individual patient data meta-analysis of four RCTs confirmed a benefit of vasopressin in addition to norepinephrine vs. norepinephrine alone in terms of renal replacement therapy requirements [73]. Although this meta-analysis did not show any difference in 28-day mortality, less 90-day mortality was observed with vasopressin in patients with normal renal function at baseline [73], a result that needs to be confirmed.

### 3.2. What Is the Optimal Time to Introduce Vasopressin during Septic Shock?

Adding vasopressin instead of escalating the dose of norepinephrine is a weak SSC recommendation with moderate quality of evidence [7]. This is explained by the fact that none of the RCTs that compared the addition of vasopressin to norepinephrine to norepinephrine alone in patients with septic shock showed survival benefits [63,72,73,74]. Nevertheless, in the Vasopressin and Septic Shock Trial (VASST), the mortality rate was lower in the vasopressin group than in the placebo group at 28 days (26.5% vs. 35.7%, *p* = 0.05) in the prospectively defined stratum of less severe septic shock (patients receiving a dose of norepinephrine <15 μg/min at randomization) [64]. This result, which needs to be confirmed in further RCTs, suggests that vasopressin should no longer be considered a rescue vasopressor therapy. Some recent data also suggest that not delaying vasopressin administration is reasonable [75,76]. In a multivariate analysis of a retrospective study, the odds of in-hospital mortality increased by 20.7% for every 10 µg/min increase in patients who received a norepinephrine-equivalent dose up to 60 µg/min at the time of vasopressin initiation [75]. No association was detected when the norepinephrine-equivalent dose exceeded 60 µg/min [75]. There was also a significant interaction between timing of vasopressin initiation and lactate concentration (*p* = 0.02) for the association with in-hospital mortality [75]. Indeed, higher norepinephrine-equivalent dose at vasopressin initiation and higher lactate concentration at vasopressin initiation were each associated with higher in-hospital mortality in patients with septic shock who received vasopressin [72].

In a multicenter retrospective cohort, the effect of early introduction of vasopressin (within 48 h of shock onset) was evaluated by a composite outcome: the proportion of patients who presented a change in the sequential organ failure assessment (SOFA) score of >3 from baseline to 72 h after initiation of vasopressin and/or in-hospital all-cause mortality [76]. The multivariate analysis showed that the time to vasopressin initiation but not the norepinephrine dose at vasopressin initiation was significantly associated with the composite outcome. For every hour delay in vasopressin initiation, the odds of the composite outcome occurring increased. In patients who received vasopressin within 7 h, there was a shorter time to hemodynamic stability, a lesser risk to develop acute kidney injury within 7 days, and a shorter time to be discharged from the intensive care unit [76].

Despite findings in favor of early administration of vasopressin, there are no data that suggest using vasopressin as the first-line vasopressor instead of norepinephrine. Doing so would not be logical since vasopressin deficiency is believed to occur 36 h from the onset of septic shock [61]. In addition, unlike norepinephrine, vasopressin does not redistribute blood from the unstressed to the stressed blood volume [77,78,79] and has no positive effect on cardiac contractility. Moreover, decreases in cardiac output were observed with vasopressin in two clinical studies [80,81]. In this regard, administration of vasopressin must be cautious if a hypodynamic shock state is suspected, so that systemic hemodynamic monitoring may be helpful, although there is no strong specific recommendation about it.

### 3.3. What Is the Suitable Dose of Vasopressin in Septic Shock?

It has been suggested that the vasopressin dose should target physiological plasma vasopressin concentrations [82]. Unlike the norepinephrine dosing, a weight-based vasopressin dosing is not necessary as body mass index does not alter the effects of vasopressin on hemodynamic stability or changes in MAP when the drug is administered as a fixed-dose infusion [83]. Continuous infusion of vasopressin at 0.03 U/min has been proposed to achieve adequate serum vasopressin concentrations and decrease catecholamine vasopressor requirements without an increase in adverse events [63]. However, the optimal vasopressin dose remains controversial. A retrospective study [84] and an RCT [85] showed that, compared with 0.03 U/min, vasopressin at a double dose (0.067 U/min) resulted in lower requirements of norepinephrine and in a better hemodynamic status in terms of MAP and lactate levels. In these two studies [84,85], there was no difference in side effects, but skin adverse effects were not reported. A high incidence (30%) of ischemic skin lesions was reported in a study where the median dose of vasopressin was 0.0009 U/kg/min, i.e., approximately 0.06–0.09 U/min for a body weight between 70 and 100 kg [86]. In the VANISH trial [72], there was no difference in the incidence of serious adverse events between vasopressin (10.7%) and placebo groups (8.3%). Interestingly, the mean vasopressin dose was 0.06 U/min and the mean norepinephrine dose was 0.79 μg/kg/min in the placebo group, at the time of onset of serious adverse events [72]. The incidence of digital ischemia was numerically but not significantly higher in the vasopressin group (5.4%) compared to placebo group (1.5%). Note that, in the VASST study, where the maximal vasopressin dose was 0.03 U/min, the incidence of digital ischemia was 2% in the vasopressin group [63].

Based on the current scientific knowledge, it is hard to have any certainty about the optimal vasopressin dose to administer. Choosing a vasopressin dose of 0.03 U/min seems reasonable insofar as the target MAP is achieved, especially if the norepinephrine dose can be reduced. Otherwise, increasing the dose up to a maximum of 0.06 U/min cannot be discouraged if the onset of adverse effects can be carefully monitored. Finally, it is noteworthy that, compared to adrenergic vasopressors, vasopressin was not associated with an increased risk of acute mesenteric ischemia in patients with septic shock, as reported by a recent meta-analysis [87].

### 3.4. Should Vasopressin Be Weaned before or after Norepinephrine?

Vasopressor therapies are titrated down and discontinued during the resolving phase, which often necessitates a decision of whether norepinephrine or vasopressin is the most appropriate as the final vasopressor.

A recent patient-level meta-analysis was performed to determine the impact of vasopressin and norepinephrine discontinuation order on clinically significant outcomes in the recovery phase of septic shock [88]. Six studies of low or moderate risk of bias with 957 patients were included [88]. Clinically significant hypotension occurred more frequently when vasopressin was discontinued first compared to norepinephrine, but without difference in mortality or lengths of stay [88].

It has been hypothesized that, when vasopressin is discontinued, hypotension occurs if the vasopressinergic system is not adequately restored [89]. Accordingly, a prospective study showed that patients who had an elevated serum copeptin concentration (a surrogate of serum vasopressin concentration) were less likely to develop hypotension when vasopressin was discontinued first [89].

However, caution is required about interpretation of these studies [88,89], due to marked heterogeneity in terms of definition of hypotension, norepinephrine doses at vasopressin discontinuation, and concomitant corticosteroids administration. Therefore, the question of which vasopressor should be weaned first is still not fully answered. The dosage of markers able to evaluate the vasopressinergic system—such as copeptin—may be helpful to make an appropriate decision. Copeptin, a 39-amino-acid glycopeptide, is the C terminal of pro-vasopressin (similar to the C peptide of insulin). It is stable in plasma and is therefore easier to measure than vasopressin. Some studies suggested that copeptin could be used as a surrogate for measurement of vasopressin [90,91]. Although copeptin correlates with vasopressin plasma concentration [92], it may be inadequate in some situations, such as in patients receiving veno–venous hemofiltration [93,94], and by consequence further evaluations are needed before deciding of its routine use.

## 4. Conclusions

Although there is no doubt that norepinephrine should be the first-line vasopressor, unresolved questions remain on the place of vasopressin. Today, addition of vasopressin rather than escalating the dose of norepinephrine makes sense, although no strong evidence exists in spite of numerous studies performed over the last years. 

Multimodal strategies, consisting of adding angiotensin 2 to norepinephrine or angiotensin 2 to the combination of norepinephrine and vasopressin [95,96], or consisting of adding methylene blue to other vasopressors [97,98], have been already tried. They generally resulted in a norepinephrine-sparing effect and/or increase in MAP but not in improved survival compared to norepinephrine alone or combined with vasopressin. However, clinical trials adequately powered to show outcome benefits are still lacking. In addition, it is likely that adding a vasopressor such as angiotensin 2 to other vasopressors would be beneficial in some specific subpopulations identified by biomarkers such as renin concentration or the angiotensin 1/angiotensin 2 ratio [99]. 

In the future, it is likely that the most appropriate vasopressors will be individually selected according to single-nucleotide polymorphism genotype biomarkers [100]. Optimal vasopressor therapy could then be a combination of agents acting on different receptors with minimizing doses of each agent and perhaps increasing safety [100].

## Figures and Tables

**Table 1:** Resolved and unresolved questions about the use of norepinephrine in septic shock.

Questions	Answers
Why is norepinephrine the recommended **first-line vasopressor** in septic shock?	Potent **vasoconstrictor** **Less** cardiac **arrhythmia** than dopamine in a RCT **Less mortality** than dopamine in meta-analyses
Should norepinephrine be administered **early** or only **after completion** of fluid resuscitation?	**Early** if vascular tone depression assumed to play a major role in hypotension Certainly, if **MAP** < **65** mmHg Certainly, if **DAP** is **low**, especially in cases of tachycardia
How should the **dose** of norepinephrine be **adapted**?	Titration according to MAP **MAP** of **at least 65** mmHg should be targeted **Higher** target **MAP** in cases of **chronic hypertension** Consider **MAP–CVP** difference if **CVP** is **high**
Should we **escalate** the doses of norepinephrine in cases of **refractory** hypotension?	**No** definitive **answer** Re-check that **source** of **infection** well **controlled** Consider infusing **hydrocortisone** after 4 h from onset of resuscitation Consider adding **vasopressin**

RCT: randomized controlled trial; MAP: mean arterial pressure; DAP: diastolic arterial pressure; CVP: central venous pressure.

**Table 2:** Resolved and unresolved questions about the use of vasopressin in septic shock.

Questions	Answers
What is the **rationale** for **adding vasopressin** to norepinephrine in some forms of septic shock?	Relative vasopressin **deficiency** in septic shock Potent **vasoconstriction** in hypotensive states **Reduction** in **norepinephrine requirement** Potential **benefits** in **renal** function
What is the optimal **time** to introduce vasopressin?	**No defined time** from the onset of resuscitation Probably, in the **first hours** rather at a late phase **No defined dose** of norepinephrine already used Probably, when the dose of norepinephrine is being **escalated**
What is the suitable **dose** of vasopressin?	A dose of **0.03 U/min** is reasonable insofar as the target MAP is achieved especially if the norepinephrine dose can be reduced. Otherwise, increasing the dose **up** to a **maximum** of **0.06 U/min** cannot be discouraged if the onset of **adverse effects** can be carefully **monitored**.
Should **vasopressin** be **weaned** before or after norepinephrine?	More frequent clinically significant **hypotension** when vasopressin is **discontinued first** Markers of the vasopressinergic system—such as **copeptin**—may help to make an appropriate decision

MAP: mean arterial pressure; DAP: diastolic arterial pressure.

## Data Availability

Not applicable.
